# Impact of parental psychosocial profile on the effectiveness of oral hygiene interventions in preschool children: a randomised controlled clinical trial in a rural population

**DOI:** 10.1007/s40368-026-01229-4

**Published:** 2026-05-27

**Authors:** Anny Caroline Schade, Karina Duarte Vilella, Erick Tassio Barbosa Neves, Ana Flávia Granville-Garcia, Fernanda de Moraes Ferreira, Luciana Reichert da Silva Assunção

**Affiliations:** 1 Federal University of Paraná, Curitiba, Brazil; 2 State University of Paraíba, Campina Grande, Brazil; 3 Federal University of Minas Gerais, Belo Horizonte, Brazil

**Keywords:** Oral hygiene, Child preschool, Rural population, Psychosocial functioning

## Abstract

**Objective:**

To assess the impact of parental psychosocial profile on the effectiveness of educational interventions aiming to improve oral hygiene among preschool children living in a rural area.

**Methods:**

A controlled randomised trial (RBR-44nrnjz, March 5, 2023) was conducted with 78 preschool children and their caregivers residing in a rural area in southern Brazil. The participants were randomly assigned to two intervention groups: standard guidance (SG) and the teach-back technique (TB). The study comprised three phases: baseline (oral examination and data collection), Phase I (educational intervention), and Phase II (oral examination seven days post-intervention). Oral hygiene was assessed using the visible plaque index (VPI) and the Simplified Oral Hygiene Index (OHI-S). For OHI-S assessment, a mature dental biofilm (> 24 h) was identified with a disclosing agent. Psychosocial factors were assessed using the sense of coherence (SOC) and parental locus of control (PLOC) instruments. For statistical analysis, participants were categorised into two clusters: *Cluster 1* (lower SOC and higher internal PLOC) and *Cluster 2* (higher SOC and lower internal PLOC). Data were analysed using non-parametric tests.

**Results:**

A reduction in the number of teeth with visible plaque was observed only among participants in *Cluster 1* subjected to the TB intervention. OHI-S scores decreased, regardless of the caregivers’ psychosocial profile or the type of intervention.

**Conclusion:**

The psychosocial profile of caregivers did not affect the effectiveness of oral health educational interventions when a mature dental biofilm was considered.

## Introduction

Dental caries is the most common oral disease in preschool children and is primarily caused by the accumulation of dental biofilm. Its prevention depends on maintaining a healthy and low-sugar diet and regular toothbrushing with fluoridated toothpaste (Chawłowska et al. 2022). Parents and caregivers play a critical role in shaping children’s oral hygiene practices and dietary behaviours, as well as in seeking dental care and making treatment decisions on their behalf (Manohar and Mani 2017). Poor oral hygiene in preschool children is associated with family-related factors such as low maternal education and low socioeconomic status (Castilho et al. 2025). Limited access to dental services in rural areas and small towns may also adversely affect children’s oral health (Camerini et al. 2020).

Recent studies have increasingly examined the influence of parental psychosocial factors, assessed using instruments such as sense of coherence (SOC) and locus of control (LOC), on children’s oral health (Tabakcilar et al. 2023; Granville-Garcia et al. 2018). SOC is a psychological construct that reflects an individual’s capacity to manage stress and cope with everyday challenges (Antonovsky 2002), whereas LOC refers to personal beliefs about who or what determines life events (Dela Coleta and Dela Coleta, xxxx). Parents with a high SOC and an internal LOC are more likely to adhere to oral health preventive guidelines and to have children with more favourable oral health outcomes (Almeida et al. 2023; Tabakcilar et al. 2023). Beyond the individual level, the existing literature suggests that geographical location (rural vs urban) may also be associated with different psychosocial profiles (Shifrer and Sutton 2014). These findings underscore the importance of considering the psychosocial characteristics of individuals or populations in intervention studies.

Patient-centred interventions aim to improve oral health outcomes by aligning care with patients’ individual needs and promoting adherence (Ceyhan et al. 2018). Among these intervention strategies, the teach-back (TB) technique has been shown to be more effective in promoting adherence to oral health instructions than more conventional approaches (Sleiman et al. 2023). This technique involves confirming a participant’s understanding by asking them to repeat the clinician’s instructions in their own words (Anderson et al. 2020). A study conducted in the USA demonstrated the effectiveness of the TB technique in educating parents of preschool children about the appropriate amount of fluoridated toothpaste to be applied to the toothbrush (Chen et al. 2018).

Few studies have evaluated the influence of psychosocial factors on the effectiveness of oral health interventions and, to our knowledge, the existing literature has focused primarily on adolescents (Potdar et al. 2015) and adults (Rawlinson et al. 2021). Despite the growing interest in the relationship between caregiver profiles and child oral health outcomes, studies examining how parental psychosocial factors influence the effectiveness of oral hygiene educational interventions in preschool children are still lacking. The hypothesis of this study is that parents or caregivers with an unfavourable psychological profile exhibit lower adherence to oral health guidelines, resulting in greater dental biofilm accumulation in preschool children.

The objective of this randomised controlled clinical trial was to assess the influence of parental psychosocial profiles on the effectiveness of interventions aimed at improving oral hygiene in preschool children living in a rural area.

## Methods

### Study population

The study was conducted in accordance with the Declaration of Helsinki and was approved by the Research Ethics Committee of the Federal University of Paraná (process no. 60163822.9.0000.0102). Written informed consent was obtained from all participants.

The reporting of this clinical trial followed the CONSORT guidelines. The study was registered at ClinicalTrials.gov on March 5, 2023, under no. RBR-44nrnjz.

This was a randomised controlled clinical trial conducted between February 2022 and May 2024, involving parent–child dyads comprising parents or caregivers and children aged 4–5 years residing in a rural area of Tijucas do Sul, state of Paraná, southern Brazil. The municipality, with an estimated population of 17 621 and a population density of 26.23 inhabitants /km^2^, is classified as predominantly rural. In 2020, the per capita income was R$ 24 601.21 and, in 2022, its human development index was 0.636 (IBGE 2022).

The following parameters were used for sample size calculation: a statistical power of 80%, a significance level of 5%, and an expected 30% difference in the mean clinical parameters between the two intervention methods evaluated in this study (Sealed Envelope Ltd 2012). A power calculator for a continuous outcome superiority trial (online) was used. Based on this calculation, the initial sample should include 72 participants. The sample size was increased by 30% to account for potential dropouts, resulting in a final target sample of 94 individuals. A parallel randomised design was utilised, with participants assigned to one of the two educational interventions in blocks of four. Randomisation was performed using a sequence of random numbers generated in Microsoft Excel. Allocation was performed by a researcher who remained blinded to the type of intervention applied.

The study included parents/caregivers aged over 18 years who were native speakers of Brazilian Portuguese, along with children aged 4–6 years who were enrolled in two local schools selected by convenience sampling. School 1 had 90 enrolled children, and School 2 had 20. Participants were randomly selected in proportion to the number of enrolled children at each school, resulting in a sample of 64 children from School 1 and 14 from School 2. Participants were excluded if they had sensory or other impairments that could interfere with the interventions, exhibited disruptive behaviour during the oral examinations, were wearing orthodontic appliances, or had diagnosed disabilities, syndromes, or neurological conditions.

### Pilot study

A pilot study was conducted with ten preschool children and their caregivers who had characteristics similar to those of the main study population. The aim of this phase was to evaluate the proposed methodology, particularly with regard to the content and language of the guidance provided. During the implementation of the interventions, it became evident that certain information required simplification to enhance clarity and accessibility for the lay public.

### Interventions and study phases

The interventions were conducted with the parents or caregivers during home visits by two previously trained researchers, using two techniques: standard guidance (SG) and the TB technique (TB). Participants were randomly assigned to one of the two intervention groups.

In both groups, the researchers used printed graphic materials developed specifically for this study. These materials served solely to support the sequence of information provided and were not distributed to the participants. The materials consisted of 14 educational laminated sheets with content based on specialised literature and organised into the following topics: the importance of primary teeth; the composition of dental biofilm; the development of early childhood caries (AAPD 2022); the relationship between diet and dental caries, with emphasis on sugar consumption (ABOPED 2021); oral hygiene, with emphasis on toothbrushing and the use of fluoridated toothpaste (ADA 2014); and the importance of regular dental visits (AAPD 2022). Each sheet contained no more than three pieces of information, in accordance with the "chunk and check" technique, which is based on presenting information in small segments to enhance comprehension (Health Literacy Place 2024).

The SG group received information through individualised verbal instructions provided by a trained researcher. The TB group received the same set of instructions as the SG group, but with the application of the TB technique. Prior to the intervention, one of the researchers (KDV) received training in the TB technique, which comprises five steps: (1) explain the information using simple language; (2) check understanding by asking the participant to explain the information in their own words; (3) clarify by identifying and re-explaining any misunderstandings; (4) re-check and re-clarify by repeating steps 2 and 3 until understanding is achieved; and (5) close the loop once all misunderstandings have been addressed (© 2018 Teachback.org). The TB cycle was repeated until the information was fully understood, with a maximum of three repetitions (Talevski et al. 2020).

In addition to the verbal instructions, a demonstration of the "back-and-forth" toothbrushing technique (Brasil 2015) was provided using a dental macromodel and a child’s toothbrush. In the SG group, only the demonstration was provided, while in the TB group, participants were asked to perform the technique following the demonstration.

**Fig. 1:**
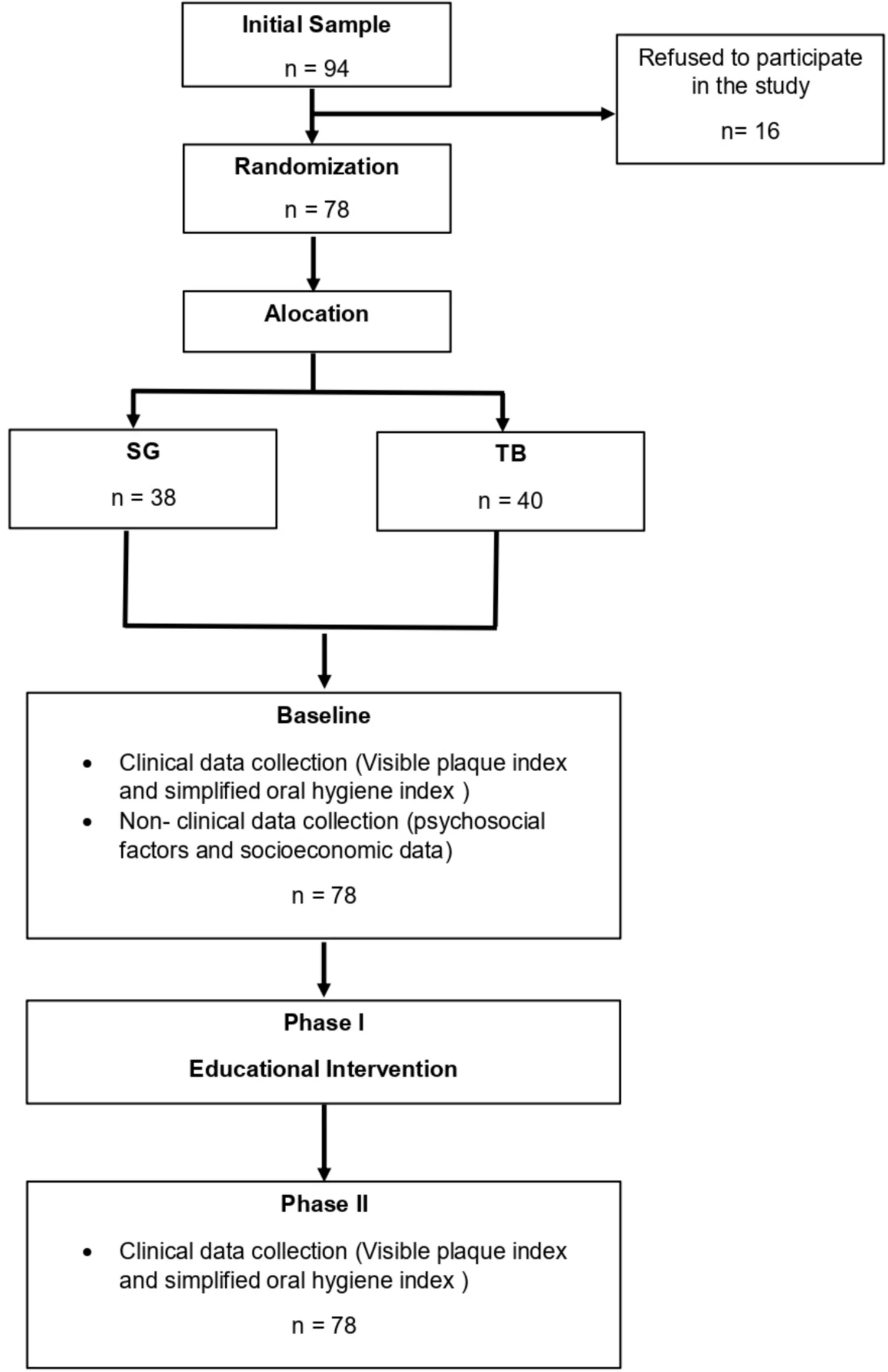
Flowchart illustrating the study phases

The study was organised into three phases: baseline, Phase I, and Phase II. At baseline, dental biofilm in the preschool children was assessed using the visible plaque index (VPI) (Löe 1967) and the Simplified Oral Hygiene Index (OHI-S) (Greene and Vermillion 1964), together with non-clinical data, including socioeconomic information and parental or caregiver psychosocial factors. In Phase I, the educational interventions were conducted, and in Phase II, which took place seven days later, the children’s clinical data were collected again. Figure 1 illustrates the flow of participants through the different phases of the study.

“Dr Dentuço” oral hygiene kits (Colgate®) were provided to all participants at baseline. Each kit included a child’s toothbrush, toothpaste containing 1 450 ppm fluoride, dental floss, and an illustrative booklet designed for children.

## Non-clinical data

### Economic and sociodemographic data

At baseline, socioeconomic and demographic data were collected from the caregiver, including age, marital status, level of education, number of residents in the household, and monthly family income. Socioeconomic status was assessed according to the criteria of the Brazilian Association of Research Companies (ABEP 2021), which assign scores based on the ownership of household items. Participants were classified into six economic categories, ranging from A to E.

### Psychosocial factors

Parental or caregiver psychosocial factors were assessed using two instruments validated in Brazilian Portuguese: the SOC scale (Bonanato et al. 2009) and the parental locus of control (PLOC) scale (Cerqueira and Nascimento 2008).

SOC refers to how individuals comprehend the human experience of life and, more specifically, the factors that promote health and well-being. The instrument comprises three dimensions: (1) comprehensibility, which concerns the perception that stimuli (whether pleasant or stressful) are understandable and predictable; (2) manageability, related to the perception that the necessary resources to face and resolve demands are available and under control; and (3) meaningfulness, which corresponds to the motivation to seek resources and face adversities (Antonovsky 2002). The SOC assessment instrument consists of 13 items, rated on a five-point Likert scale, with total scores ranging from 13 to 65 (Bonanato et al. 2009).

PLOC, in turn, is assessed using an 18-item instrument distributed across three subscales: internal, powerful others, and chance. These dimensions indicate whether individuals attribute life outcomes to themselves (internal), to people in positions of authority (powerful others), or to random factors such as fate (chance). Each subscale contains six items, with five response options ranging from 1 (strongly agree) to 5 (strongly disagree), yielding scores ranging from 6 to 30 for each dimension (Cerqueira and Nascimento 2008). Participants were categorised as internal if their total score was lower on the internal subscale, and as external if their total score was lower on the external and chance subscales (Gomes et al. 2022).

### Clinical data

Dental biofilm was assessed by two calibrated examiners (kappa ≥ 0.80) using two different indices: the VPI (Löe 1967) and the OHI-S (Greene and Vermillion 1964). The buccal surfaces of teeth 52, 51, 61, and 62 were evaluated for VPI but, in the absence of these teeth, their permanent successors were evaluated (Alaluusua and Malmivirta 1994). Subsequently, the same surfaces were stained using a two-colour plaque disclosing solution (Angie^®^, Angelus, Londrina-PR, Brazil). According to the manufacturer, blue indicated mature plaque (> 24 h), whereas pink indicated more recent plaque (< 24 h). The blue-stained surface was assessed and scored on a four-level scale: 0 (no staining), 1 (one-third of the tooth surface), 2 (two-thirds of the tooth surface), and 3 (the entire tooth surface). The scores for each participant were then summed and divided by the number of teeth assessed to calculate the OHI-S (Greene and Vermillion 1964).

The assessments were conducted in the school environment, without prior notification to parents or caregivers regarding the assessment day. Examinations were conducted under natural light at a standardised time, before the school snack. Biofilm assessments were performed at baseline (prior to the interventions) and seven days later (Phase II).

The children were blinded to the type of intervention received by their caregivers.

### Statistical analysis

Descriptive statistics for numerical variables were expressed using measures of central tendency and variability, while categorical variables were reported as absolute and relative frequencies. Categorical variables were analysed using the Chi-square test or Fisher’s exact test.

Numerical variables were tested for normality using the Kolmogorov–Smirnov test. If the variable presented a symmetrical distribution, Student’s t-test for independent samples or paired Student’s t-test was used, as appropriate. For asymmetrically distributed data, the Mann–Whitney *U* test was applied for comparisons between independent groups, while the Wilcoxon signed-rank test was used for paired groups.

Cluster analysis was performed to categorise participants into groups characterised by internal homogeneity and external heterogeneity, based on psychosocial factors. SOC and PLOC were selected for this analysis because of their similar conceptual framework in behavioural constructs (Nowicki et al. 2017). Prior to the analysis, participants were categorised based on their median scores on the two instruments: lower SOC (< 34) and higher SOC (≥ 35), and less internal PLOC (< 10) and more internal PLOC (≥ 10). Only scores from the internal dimension of the PLOC were considered.

A two-step cluster analysis was performed, resulting in two distinct clusters: "Cluster 1"—lower SOC and higher internality in PLOC, and "Cluster 2"—higher SOC and lower internality in PLOC. The quality of the cluster solution was assessed using different statistical and conceptual criteria. The silhouette measure indicated good internal cohesion and separation between clusters (coefficient > 0.5), while the Bayesian information criterion was used to determine the optimal number of clusters, showing a favourable inflection point in the two-cluster solution. Additionally, cluster sizes were balanced, with no evidence of artificial groupings or outliers. External validity was reinforced by comparing the groups on variables not included in cluster formation, using appropriate statistical tests. Finally, the theoretical interpretability of the clusters was consistent with the adopted framework, lending robustness to the obtained segmentation. The PLOC demonstrated greater predictive power in cluster formation compared with the SOC.

All analyses were performed using SPSS for Windows software (version 22.0; IBM Corp., Armonk, NY, USA), with a significance level set at 5%.

## Results

At baseline, 94 parent–child dyads were selected; however, 12 declined to participate, resulting in a total of 78 dyads.

Table 1 presents the baseline characteristics of the study population. Most participants were mothers, who had more than eight years of formal education and belonged to socioeconomic class C or lower. The mean number of teeth with biofilm, as assessed by the VPI, was 2.95 in the SG group and 3.30 in the TB group. The mean OHI-S score was 1.93 in the SG group and 1.49 in the TB group. The intervention groups were homogeneous for all socioeconomic, demographic, psychosocial, and clinical variables at baseline (p > 0.05).

**Table 1:** Characteristics of the study population (baseline)

Variables	SG	TB	p-value
Caregiver’s age—mean (SD)	31.11 (5.75)	32,48 (5,89)	0.305****
Relationship to the child (*n*, %)			
Mother	32 (45.07)	39 (54.93)	
Father	2 (100.0)	0 (0.0)	
Others	4 (80.0)	1 (20.0)	
Child’s sex (*n*,%)			
Male	17 (51.52)	16 (48.54)	0.672*
Female	21 (46.65)	24 (53.43)	
Economic class (*n*,%)			
≥ B	5 (71.50)	2 (28.55)	0.257**
≤ C	33 (46.52)	38 (53.55)	
Monthly per capita income in BRL- mean (SD)	675.50 (287.27)	655.21 (340.60)	0.663****
Caregiver’s education (*n*,%)			
> 8 years	24 (72.71)	26 (70.27)	0.820*
≤ 8 years	9 (27.24)	11 (29.79)	
Caregiver’s marital status (*n*, %)			
Married or cohabiting	29 (48.32)	31 (51.42)	0.901*
Single/separated/widowed	9 (50.0)	9 (50.0)	
SOC (median; min–max)	35.32 (23–46)	32.51 (17–46)	0.265***
Internal LOC (*n*, %)	36 (50.0)	36 (50.0)	0.433**
External LOC (*n*,%)	2 (33.32)	4 (66.71)	
Internal PLOC (median; min–max)	10.0 (6–16)	12.31 (6–18)	0.236***
Number of teeth with visible plaque (mean; SD)	2.95 (1.11)	3.30 (0.97)	0.153****
OHI-S (mean; SD)	1.93 (0.68)	1.49 (0.62)	0.348****

Values below 78 are due to missing data for the variable

^*^ Chi-square test; ** Fisher’s exact test; *** Student’s t-test for independent samples, **** Mann–Whitney *U* test

*SG* standard guidance, *TB* teach-back, *SD* standard deviation, *SOC* sense of coherence, *LOC* locus of control, *PLOC* parental locus of control, *OHI*-*S* Simplified Oral Hygiene Index

Considering the total sample, regardless of psychosocial profile and type of intervention, a reduction in the number of teeth with visible plaque was observed in Phase II compared with baseline (p = 0.012). Similarly, OHI-S scores were significantly lower in Phase II than at baseline (p < 0.001).

Cluster 1 consisted of 34 participants and Cluster 2 of 44 participants. Among the 34 parents/caregivers in Cluster 1, 15 (44.1%) were assigned to SG and 19 (55.9%) to TB. Among the 44 parents/caregivers in Cluster 2, 23 (52.3%) were assigned to SG and 21 (47.7%) to the TB technique. At baseline, no significant difference was observed in the distribution of participants across clusters and types of intervention (P = 0.475).

Table 2 shows the comparison of the number of teeth with visible plaque, as assessed by the VPI, according to the type of intervention at baseline and Phase II, stratified by psychosocial clusters (Cluster 1 and Cluster 2). In Cluster 1 (lower SOC and more internal LOC), the TB group presented a higher number of teeth with visible plaque compared with the SG group at baseline. In the within-group comparison, a reduction in the number of teeth with visible plaque was observed only in the TB group. In Cluster 2, no significant differences were observed in between-group or within-group comparisons (p > 0.05) for either intervention (TB and SG).

**Table 2:** Assessment of the number of teeth with visible plaque (VPI) according to the type of intervention at baseline and Phase II, stratified by psychosocial clusters (Cluster 1 and Cluster 2)

Group	Baseline mean (SD)	Phase II mean (SD)	Mean difference	95% CI	p-value*
Cluster 1					
TB	3.42 (1.02)	2.95 (1.22)	0.47	(0.07 to 0.88)	**0.030**
SG	2.53 (1.18)	2.40 (1.50)	0.13	(−0.65 to 0.91)	0.793
p-value**	**0.027**	0.319			
Cluster 2					
TB	3.19 (0.93)	2.67 (1.15)	0.52	(−0.13 to 1.18)	0.124
SG	3.22 (0.99)	2.78 (1.12)	0.44	(−0.23 to 1.10)	0.170
p-value**	0.817	0.797			

TB = Orientation with *teach-back* technique

^*^Wilcoxon test (within-group comparison); **Mann–Whitney *U* test (between-group comparison)

Statistically significant values are highlighted in bold

*TB* teach-back, *SG* standard guidance, *SD* standard deviation

Table 3 shows the comparison of OHI-S scores according to the type of intervention at baseline and Phase II, stratified by psychosocial clusters (Cluster 1 and Cluster 2). Cluster 1 revealed a significant reduction in scores in Phase II compared with baseline in the within-group analysis for both interventions. Conversely, in the between-group analysis, no significant differences in OHI-S were observed when comparing the two phases of the study. Cluster 2 showed a significant reduction in scores in Phase II compared with baseline in the within-group analysis for both interventions. In the between-group analysis, no significant differences were observed when comparing the two phases of the study (p > 0.05).

**Table 3:** Assessment of the Simplified Oral Hygiene Index (IHO-S) according to the type of intervention at baseline and Phase II, stratified by psychosocial clusters (Cluster 1 and Cluster 2)

Group	Baseline mean (SD)	Phase II mean (SD)	Mean difference	95% CI	p-value*
Cluster 1					
TB	1.58 (0.67)	1.31 (0.66)	0.27	(0.10 to 0.44)	**0.005**
SG	1.22 (0.54)	1.01 (0.68)	0.21	(−0.03 to 0.44)	**0.042**
p-value**	0,051	0,242			
Cluster 2					
TB	1.41 (0.58)	1.09 (0.70)	0.32	(0.09 to 0.54)	**0.011**
SG	1.50 (0.74)	1.12 (0.58)	0.38	(0.07 to 0.48)	**0.014**
p-value**	0.896	0.320			

^*^Wilcoxon test (within-group comparison); **Mann–Whitney *U *test (between-group comparison)

Statistically significant values are highlighted in bold

*TB* teach-back, *SG* standard guidance, *SD* standard deviation

The within- and between-group comparisons between clusters, stratified by the type of intervention, are presented in Tables 4 and 5. A significant within-group difference (Baseline–Phase II) was observed in Cluster 1 for the mean number of teeth with visible plaque among participants who received the TB intervention. No significant differences were observed between clusters at either phase (Table 4). For the OHI-S, no within- or between-cluster differences were found at baseline or Phase II (Table 5).

**Table 4:** Assessment of the number of teeth with plaque using the Visible Plaque Index (VPI) according to psychosocial clusters (Cluster 1 and Cluster 2) at baseline and Phase II, stratified by the type of intervention

Group	Baseline mean (SD)	Phase II mean (SD)	Mean difference	95% CI	p-value*
TB					
Cluster 1	3.42 (1.02)	2.95 (1.22)	0.47	(0.07 to 0.88)	**0.030**
Cluster 2	3.19 (0.93)	2.67 (1.15)	0.52	(−0.13 to 1.18)	0.124
p-value**	0.333	0.405			
SG					
Cluster 1	2.53 (1.18)	2.40 (1.50)	0.13	(−0.65 to 0.91)	0.793
Cluster 2	3.22 (0.99)	2.78 (1.12)	0.44	(−0.23 to 1.10)	0.170
p-value**	0.095	0.497			

^*^Wilcoxon test (within-group comparison); **Mann–Whitney *U *test (between-group comparison)

Statistically significant value is highlighted in bold

*TB* teach-back, *SG* standard guidance, *SD* standard deviation

**Table 5:** Assessment of the Simplified Oral Hygiene Index (IHO-S) according to psychosocial clusters (Cluster 1 and Cluster 2) at baseline and Phase II, stratified by the type of intervention

Group	Baseline mean (SD)	Phase II mean (SD)	Mean difference	95% CI	p-value*
TB					
Cluster 1	1.58 (06.77)	1.31 (0.66)	0.27	(0.10 to 0.44)	**0.005**
Cluster 2	1.41 (0.58)	1.09 (0.70)	0.32	(0.09 to 0.54)	**0.011**
p-value**	0.161	0.187			
SG					
Cluster 1	1.22 (0.54)	1.01 (0.68)	0.21	(−0.03 to 0.44)	**0.042**
Cluster2	1.50 (0.74)	1.12 (0.58)	0.38	(0.07 to 0.48)	**0.014**
p-value**	0.497	0.391			

^*^Wilcoxon test (within-group comparison); **Mann–Whitney *U *test (between-group comparison)

Statistically significant values are highlighted in bold

*TB *teach-back, *SG *standard guidance, *SD *standard deviation

## Discussion

The hypothesis of this study was confirmed for only one of the clinical indices used to assess dental biofilm control. A reduction in the number of teeth with visible plaque in Phase II compared with baseline was observed only among participants with lower SOC/more internal LOC (Cluster 1) who received instructions through the TB technique (Table 2). The superiority of the TB group over the SG group in Cluster 1 was also evident in the lack of statistical significance in the between-group comparison in Phase II. Although the SG group had a lower mean number of teeth with visible plaque at baseline, it did not show a significant reduction compared with the TB group in Phase II.

This pattern was also observed when analysing the results between clusters, stratified by type of intervention, with improvement in visible plaque control occurring exclusively among participants in Cluster 1 who received the TB intervention (Table 4). This finding supports the notion that individuals with a more internal LOC may respond more effectively to patient-centred communication strategies that require active engagement (Rodriguez et al. 2023), such as the TB technique. The absence of between-cluster differences in both phases suggests that the psychosocial profile did not influence baseline oral hygiene behaviour, but rather modulated the response to this specific educational approach.

These findings may be explained by the fact that LOC was the factor with the greatest predictive power in cluster formation. Individuals with a more internal LOC are perceived as taking greater responsibility for their children’s health and are more likely to value and adhere to preventive health guidelines (Nowicki et al. 2017). In contrast, parents or caregivers with a lower internal LOC tend to attribute health outcomes to external factors such as luck or fate, and are less likely to engage actively in their children’s health care (Cerqueira and Nascimento 2008).

To our knowledge, few studies have investigated the influence of LOC on children’s oral hygiene practices (Vanagas et al. 2009), with more recent research focusing on caries experience (Nunes and Peroza 2017; Kateeb et al. 2023). A previous study conducted in Brazil reported reduced caries experience in preschool children whose parents or caregivers had higher internal PLOC scores (Nunes and Peroza 2017). Similar findings were reported in a study of Palestinian preschool children living in a province of Jerusalem, in which parents with a higher internal LOC were associated with a lower incidence of dental caries in their children (Kateeb et al. 2023). When caregivers do not perceive themselves as co-responsible for their child’s oral health care, the risk of caries in primary teeth may increase, possibly because they defer preventive care to others, resulting in delayed intervention (Nunes and Peroza 2017).

This is the first study to assess the influence of the parental psychosocial profile on the effectiveness of patient-centred and more conventional oral health-related educational approaches. Although TB is widely used in other health fields, data on its application in dentistry remain limited. A study conducted in the USA with parents or caregivers of preschool children revealed greater effectiveness of the TB technique compared with the use of videos in changing oral hygiene practices (Chen et al. 2018). Organisations such as the American College of Surgeons, American Hospital Association, Agency for Healthcare Research and Quality, and the Institute for Healthcare Improvement recommend the use of the TB technique to facilitate patient understanding and adherence to treatment, and to reduce dental emergencies and appointment cancellations (Sleiman et al. 2023).

Individuals from Cluster 1 were those who, in addition to a higher internal LOC, also had lower SOC scores. Studies evaluating the influence of parental SOC on children’s oral hygiene have not yet been reported. Previous research has observed that children whose parents have higher SOC scores tend to have fewer decayed, missing, or filled teeth (Torres et al. 2020). Conversely, lower maternal SOC scores are associated with less favourable oral health behaviours and less frequent use of dental care by children (Perazzo et al. 2017). Given that SOC is linked to the ability to cope with more stressful outcomes (Kenne Sarenmalm et al. 2013), and that biofilm control is more closely related to preventive practices, this may explain the difference in findings observed in this study compared with those reported in the literature.

On the other hand, both the TB technique and more conventional oral instruction were equally effective in reducing dental biofilm in preschool children, as assessed by the OHI-S, regardless of the parental psychosocial profile (Table 3). The OHI-S is considered more specific in visualising dental biofilm than other indices, such as the VPI, as it takes into account both the location and the amount of biofilm, allowing for a more detailed assessment of biofilm accumulation (Fischman 1986). In addition, a more mature biofilm (> 24 h) was used as a parameter, given that recent evidence indicates that biofilm maturation occurs within 24 h, leading to intense acidification and increased virulence, as demonstrated by metagenomic analysis (Edlund et al. 2018).

The VPI, although more sensitive in detecting the presence of visible plaque, has lower specificity compared with the OHI-S because it does not account for the duration of biofilm presence or areas with less visible plaque, resulting in lower accuracy in identifying actual oral health status (dos Santos and Soviero 2006). Therefore, it is important that studies using interventions for dental biofilm control consider the characteristics and specificities of the clinical indices used when interpreting the results.

Some studies suggest that, in certain rural populations, there may be a tendency towards a more external LOC, in which individuals attribute the outcomes of their efforts to external factors such as luck, fate, or the influence of others, rather than to their own actions (Shifrer and Sutton 2014). In the present study, however, only 6 out of the 78 participants demonstrated scores indicative of a predominantly external LOC. This finding underscores the complexity of the perception of control, a multifactorial phenomenon shaped by sociocultural, psychological, and environmental factors (Rodriguez et al. 2023).

This study has some limitations. The sample was restricted to a rural population with specific characteristics, and schools were selected by convenience sampling, limiting external validity and generalisability to populations with different socioeconomic and cultural backgrounds. Additionally, in this small rural community, communication between parents or caregivers from different groups could not be completely ruled out, potentially leading to contamination. The follow-up period may have been insufficient to observe significant long-term changes in biofilm control. Future studies with longer follow-up periods are needed for a more consistent evaluation of the impact of the psychosocial profile on the effectiveness of interventions. However, as the intervention implemented in this study was not designed to modify psychosocial factors, which are complex and shaped by various aspects of participants’ lives, the findings are unlikely to change over time.

Despite these limitations, this study makes a significant contribution, demonstrating that, regardless of the parental psychosocial profile, interventions with different approaches (one more patient-centred and the other more conventional) were equally effective in reducing dental biofilm in preschool children. Nevertheless, it is important to promote caregiver engagement in the management of children’s oral health through more personalised interventions tailored to individual and family contexts. Furthermore, future studies should explore additional oral health outcomes, including diverse populations, to further elucidate the impact of parental or caregiver psychosocial factors on the effectiveness of educational interventions.

## Data Availability

The data that support the findings of this study are not openly available due to reasons of sensitivity and are available from the corresponding author upon reasonable request.
